# Characterization of Pulmonary Bacteriobiota in Critically Ill Patients in Southern Peru through Next-Generation Sequencing Technology

**DOI:** 10.3390/cimb45120627

**Published:** 2023-12-13

**Authors:** Katherine Quispe-Medina, Jani Pacheco-Aranibar, Angel Mamani-Ruelas, Carlos Gamez-Bernabé, Rosemary Zapana-Begazo, Ivan Paz-Aliaga, Jose Villanueva Salas, Julio C. Bernabé-Ortiz

**Affiliations:** 1 Post-Graduate School, Universidad Católica de Santa María, Urb. San José s/n, Umacollo, Arequipa 04013, Peru; katherine.quispem@ucsm.edu.pe (K.Q.-M.); rzapana@ucsm.edu.pe (R.Z.-B.); jvillans@ucsm.edu.pe (J.V.S.); 2 Department of Molecular Biology, Instituto de Biotecnología del ADN Uchumayo, Arequipa 04400, Peru; jparanibar@hotmail.com; 3 Emergency and Critical Care Department, Hospital Nacional Carlos Alberto Seguín Escobedo—EsSalud, Calle Peral s/n, Arequipa 04001, Peru; amamanir@ucsm.edu.pe; 4 Human Medicine School, Faculty of Health Sciences, Universidad Nacional Jorge Basadre Grohmann, Av. Miraflores s/n, Tacna 23001, Peru; carlosgamber@hotmail.com; 5 Department of Pharmacy, Biochemistry and Biotechnology, Universidad Católica de Santa María, Urb. San José s/n, Umacollo, Arequipa 04013, Peru; ipazaliaga@ucsm.edu.pe

**Keywords:** bacteriobiota, pulmonary microbiome, critically ill patient, next-generation sequencing

## Abstract

Sequence variation in the *16S* gene is widely used to characterize diverse microbial communities. This was the first pilot study carried out in our region where the pulmonary microbiota of critically ill patients was investigated and analyzed, with the aim of finding a specific profile for these patients that can be used as a diagnostic marker. An study of critical patients mechanically ventilated for non-respiratory indications, in a polyvalent intensive care unit, was carried out; samplee were extracted by endotracheal aspiration and subsequently the microbiota was characterized through Next-Generation Sequencing Technology (NGS). The predominant phyla among the critically ill patients were Proteobacteria, Firmicutes and Bacteroidata. In the surviving patients group, the predominant phyla were Proteobacteria, Bacteroidata and Firmicutes, in the group of deceased patients thy were Firmicutes, Proteobacteria, and Bacteroidata. We found a decrease in commensal bacteria in deceased patients and a progressive increase in in-hospital germs.

## 1. Introduction

A critically ill patient has a high morbi-mortality due to a series of clinical events that complicate their evolution over the course of several days, systems can become dysfunctional leading to multi-organ failure and subsequent progression to death if the problems are not managed in time. Lower respiratory tract infection is a major contributing factor to respiratory failure requiring intubation and mechanical ventilation, which although life-saving, is at the same time one of the leading causes of complications and mortality in these patients.

In the last two decades, the study of the bacterial microbiome in the human body has been increasingly investigated, as well as its relationship with various diseases and the consequences of its alteration. The lung microbiota, however, has been studied less frequently due to its complexity of isolation and low population in the airways.

Several studies have shown that the pulmonary microbiota includes bacteria as a normal part of its flora. These limited studies, due to ethical factors, have been demonstrated using bronchoalveolar lavage (BAL) techniques, bronchoscope brushing, and swabs. The limitations of these studies remain considerable despite the current advanced techniques for the isolation and molecular characterization of microorganisms [1,2].

The pulmonary microbiota resembles the oropharyngeal microbiota, which makes it possible to affirm that this is the main source of germs in the lower airway due to the frequent microaspirations documented in healthy individuals and the fact that they are more frequent in various respiratory diseases [3,4]. It is difficult to define the bronchial microbiota of healthy individuals due to the possible effect of upper airway contamination [5].

According to studies of the central lung microbiota of non-malignant tissue, it is composed mainly of the Proteobacteria, Firmicutes, Bacteroidetes and Actinobacteria phyla and the *Proteobacteria*, *Acinetobacter*, *Pseudomonas* and *Rasltonia* genera [6].

The composition of the lung microbiome is determined, according to first principles, by the balance of three factors: microbial immigration into the airways, the removal of microbes from the airways, and the relative reproduction rates of members of their community [7], as determined by regional growing conditions. Any change in the microbiome, within an individual or across disease states, must be due to some disturbance in these factors. Sources of microbial immigration include inhalation of air containing 10^4^–10^6^ bacteria/mL even before reaching the bacteria-dense upper respiratory tract [8], subclinical microaspiration of upper respiratory tract contents, and direct dispersal along the mucosa of the airways [9,10].

Microbial clearance is driven by mucociliary clearance, (coughing—which is common even among healthy subjects) and host immune defenses (both innate and adaptive) [11].

Therefore, the main determinant of lung microbiome health is the balance between immigration and clearance [12,13].

Mechanical ventilation is a method of life support used to allow the patient more time to recover from diseases of intra or extrapulmonary origin that compromise respiratory function. Normally the human body, under healthy conditions, exchanges gases at the alveolar level, obtaining oxygen from the atmosphere through muscular work and a combination of the elastic recoil of the lung tissue and the alveolar surface tension in the expiratory phase.

The lung microbiota is not isolated; therefore, when there is microbiome alteration, whether oropharyngeal or visceral, it could increase the risk of hospital-acquired pneumonia or pneumonia associated with mechanical ventilation [14].

One of the ways to establish or reconstitute the balance of the lung microbiome is the use of probiotics when the administration of antibiotics is discontinued [15]. This was studied in murine models in which rats were fed *Lactobacillus* before being inoculated with *Streptococcus pneumoniae* resulting in the stimulation of IgG and IgA production, as well as a balance between Tumor necrosis factor (TNF) and Interleukin 10, and thus stimulating a positive immune response [16].

Studying this relationship remain challenging due to the complexity of analysis and characterization of microorganisms that make up this flora, despite the existence of techniques such as Next-Generation Sequencing.

Mechanical ventilation can alter the balance of the microbiome of both the upper and lower airways, and this may be due to multiple factors such as the presence of an endotracheal tube, inability to cough reflex, and mucociliary alteration, reducing bacterial presence [17].

The screening process to obtain and characterize the microbiome is carried out by bronchoalveolar lavage, a technique used by Smith et al., 2016, in a study of mechanically ventilated patients which, identified microorganisms such as *Hydrogenophaga*, *Bacteroides* diversity, *Pedobacter*, *Thauera* and *Acinetobacter*. These results were representative of nonpathogenic microbiomes common among these patients [18].

A hypothesis put forward by Coburn et al., 2015 suggest that bacteria that are independent of the microbiome in a concurrency network could become potential pathogens, as was observed in cystic fibrosis. These authors also observed that patients under mechanical ventilation presented an abundance of bacteria of the genera *Burkholderia* and *Bacillales* [19].

One of the consequences of this dysbiosis in the lung microbiota due to the presence of *Pseudomona aeruginosa* is a negative evolution and low chances of recovery [20].

The dynamics of the entire microbial populations in the respiratory tract of the intubated patient remain poorly understood. Current diagnostic approaches are based on the identification of causative pathogens through culture, however traditional cultures are often unable to identify all bacteria in bronchoalveolar lavage samples, in addition to requiring prolonged incubation periods. Major advances in Next-Generation Sequencing (NGS) have allowed the identification of pathogens independently of conventional microbiological culture, which has expanded research into the human lung microbiome and has many potential advantages in critically ill individuals.

The relationship between the microbiota and mechanical ventilation is fundamental and little studied; we investigated the pulmonary bacteriobiota in critically ill patients in southern Peru through NGS technology with the aim of finding a specific profile of these patients that is useful as a diagnostic marker.

## 2. Materials and Methods

### 2.1. Study Population

This prospective, observational study included a total of 12 patients subjected to mechanical ventilation admitted to a multipurpose emergency intensive care unit at the Carlos Alberto Seguin Escobedo National Hospital of the Arequipa Healthcare Network of EsSalud between October 2021 and November 2022. The exclusion criteria included patients readmitted to the ICU or referred from other medical centers, immunocompromised patients and patients with COVID-19 or with an incomplete clinical history at the time of admission, or whose family members refused to participate in the study. Clinical demographic data and parameters for SOFA and APACHE II score prognostic scales were collected at patient admission to the unit, and epidemiological data such as duration of MV, and survival at 7 and 28 days were recorded based on the electronic medical record. In addition, the results of bacterial cultures performed in the microbiology laboratory of the hospital center were considered.

This study was part of the wider study of the pulmonary microbiome promoted by Universidad Católica Santa María and Fondecyt-World Bank, which has the approval of the Research Ethics Committee of the Red Asistencial Arequipa—EsSalud, according to Directive N°03-IETSI-ESSALUD 2019 “Directive that regulates the development of Health Research EsSalud”, and with letter N° 32-UCID-GRRAR-ESSALUD-2020. Informed consent was obtained from the patient’s direct family member and dependent.

### 2.2. Sample Collection

Bronchial secretion samples were collected through an endotracheal tube secretion aspiration technique, after 100% oxygenation, using a sterile aspiration probe connected to a 40 mL busse bronchial secretion collector. The samples were collected aseptically by the investigator, part of the sample was sent for conventional culture to the hospital’s microbiology laboratory and the other part was transferred under refrigeration to the research laboratory of the Graduate School of the Universidad Católica Santa María for DNA extraction.

### 2.3. DNA Extraction and Amplification

DNA was extracted using the phenol-chloroform-isoamyl alcohol technique. Briefly, samples were removed from storage and 1 mL was passed to 1.5 mL polypropylene microcentrifuge tube, 100 µL was added, along with 100 of µL of glass microbeads, and 700 mL of cell lysis buffer (10 mM Tris—HCL, 10 mM EDTA, 50 mM NaCl, 5% SDS, pH 7.5) with 10 μL of proteinase K. The mixture was vortexed for 5 min to promote the lysis of bacteria and then incubated for 30 min at 60 °C in a water bath, before, 25 μL of RNAse was added and it was further incubated at 37 °C for 30 min, after centrifugation the supernatant was used to precipitate the DNA using isoamyl alcohol. The DNA pellet was resuspended with 50 µL molecular grade water and was kept at −20 °C. The purity of the DNA was verified in agarose gel at 1.5%, and finally the DNA was visualized in the transilluminator for electrophoresis.

### 2.4. Sequencing of the V3–V4 Region of the 16S rRNA Gene

Sequencing libraries were generated using the NEBNext^®^ Ultra^TM^ DNA Library Pre Kit for Illumina (Ipswich, MA, USA), following the manufacturer’s recommendations, and index codes were added. The quality of the library was assessed with the Qubit@ 2.0 fluorometer (Thermo Scientific, Waltham, MA, USA) and the Agilent Bioanalyzer 2100 system. Finally, the library was sequenced on an Illumina MiSeq platform and 250 bp paired-end reads were generated.

#### 2.4.1. Bioinformatic Analyses

Bioinformatic analysis were carried out as described in the Section 2 Material and Methods of the paper [21]. Briefly, the paired-end reads were merged using FLASH v. 1.2.7, and quality filtering for the raw labels was performed according to the QIIME quality control process. Sequence analysis was performed with the Uparse software (Uparse was implemented as a command in USARCH, version is 11.0.667). For each representative sequence, the GreenGene database was used to study the phylogenetic relationship of different operational taxonomic units (OTUs) and the difference between the dominant species in different samples (groups). Sequence alignment was performed with the MUSCLE software (MUSCLE is available as a free web service on the EBI website and does not have a version number: https://www.ebi.ac.uk/Tools/msa/muscle/, accessed on 1 September 2023). Alpha diversity and Beta diversity in weighted and unweighted UniFrac were calculated using QIIME software (QIIME 2 q2studio-2022.8.0).

#### 2.4.2. Statistical Analyses

Statistical analysis were performed with R software version 2.15.3, using the FactoMineR package (Vienna, Austria) and ggplot2 for principal component analysis (PCA) and cluster analysis. Principal coordinate analyses (PCoA) were performed with the WGCNA, stat, and ggplot2 packages included in the R software, For the analysis of correlations between data, Spearman’s correlation was used, which is more appropriate for measurements taken from ordinal scales such as scores (SOFA prognostic scales and APACHE II score). In this analysis the correlation coefficients were between −1 and 1, where −1 indicated a strong negative correlation and 1 a strong positive correlation. The significance value was 0.05. The graphical representation of this analysis was presented as correlograms, where the correlation coefficients were colored by value, with positive correlations shown in blue and negative correlations in red. The intensity of the color and the size of the circle were proportional to the correlation coefficients (on the right side of the correlogram, the color of the legend shows the correlation coefficients).

Finally, in order to analyze the association between the variables and mortality, the Mann-Whitney U statistical tests were used for the quantitative variables and the chi-square statistical test for the qualitative variable of age. The quantitative data were expressed in medians, while the qualitative data were expressed in percentages. The significance level was 95%, which in terms of probability ranslated into *p* = 0.05.

## 3. Results

A total of 12 mechanically ventilated critically ill patients were enrolled; their demographics characteristics are presented in Table 1. Patients were a mean of 65.8 years of age and had an Acute Physiology and Chronic Health Evaluation (APACHE II score of 20.5). A total of 6/12 (50%) were female Table 1). Patients were admitted to the intensive care unit (ICU) for surgical conditions (*n* = 6, 50%), traumatic (*n* = 5, 41.6%) and medical conditions (*n* = 1, 8.4%). The median ICU stay was 8 days and four (33.3%) patients were alive at discharge from the unit.

### 3.1. Central Microbiome and Hierarchical Clustering

**Figure 1:**
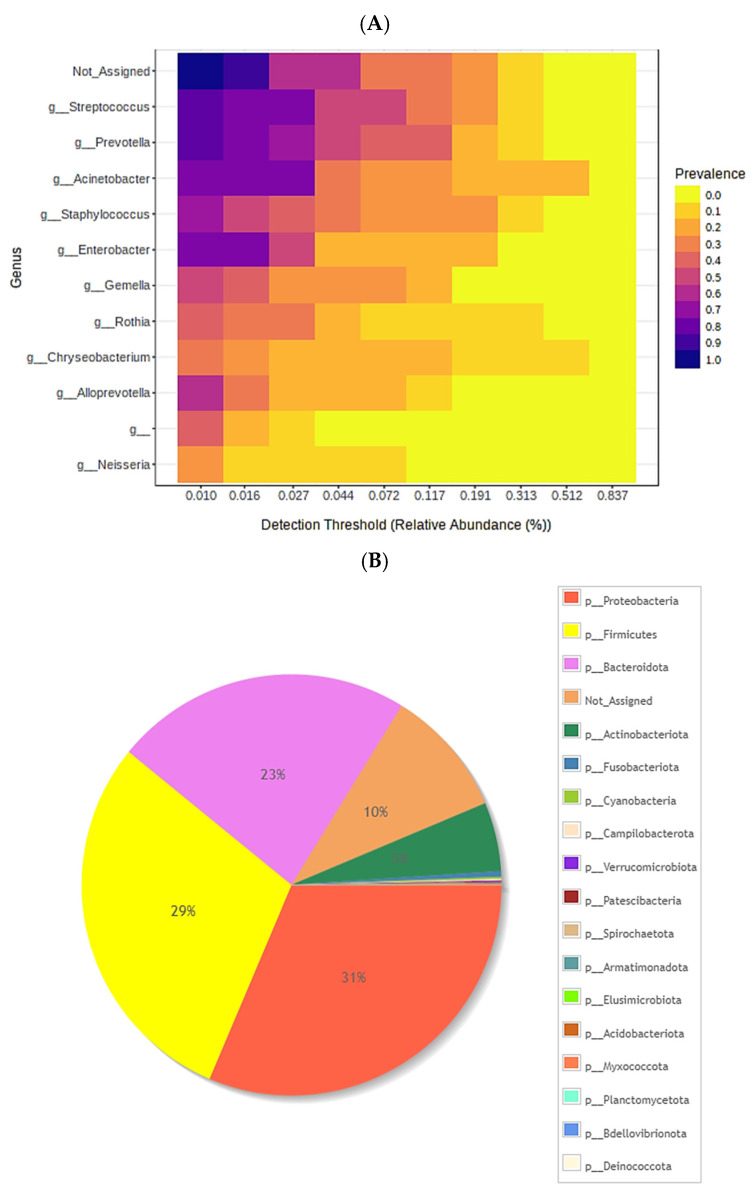
(**A**) Heat map representing the core microbiome at the genus level. (**B**) Taxonomic composition of the community at the phylum level, The other phyla (6–18) in the right column correspond to 2% of the microbiota, among the most representative. presented using a pie chart.

In the analysis of the central microbiome, the prevalence and relative abundance at the genus level were evaluated in parameters from 0 to 1 (where 1 was more prevalent). As can be seen at the general level, most were not assigned, and of those that were assigned, *Streptococcus*, *Prevotella* and *Acinetobacter* prevailed. The least present were *Alloprevotella* and *Neisseria* (Figure 1A).

In addition, it was observed that the composition at the phylum level was dominated by Proteobacteria, Firmicutes and Bacteroidata (31.29 and 23% respectively), and the least abundant phylum were Planctomycetota, Bdellovibrionota and Deinococcota (Figure 1B)

Our study considered three time slices, at 24, 48 and 72 h of intubation with the intention of assessing changes in the lung microbiota, as well as making distinctions between groups and analyzing alpha and beta diversities in both deceased and surviving patients. This analysis methodology by time cuts, as well as the method for obtaining the sample, was similar to that carried out by Kelly et al. [22].

**Figure 2:**
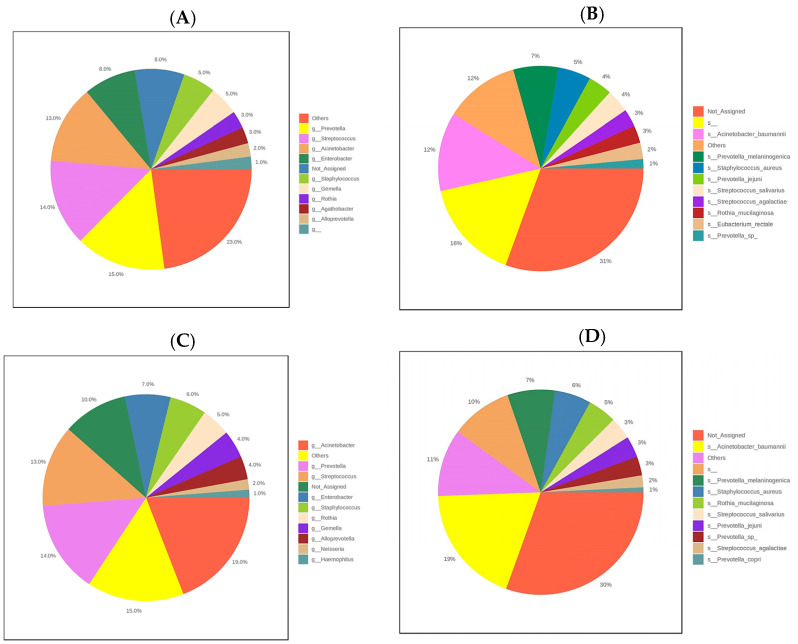

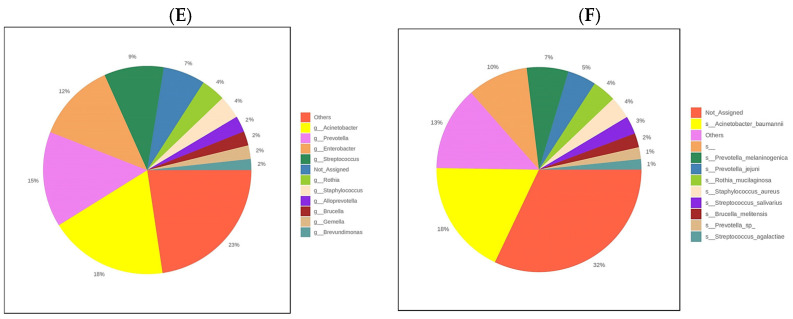
Taxonomic composition of the community. At 24 h (**A**,**B**), at 48 h (**C**,**D**) and at 72 h. (**E**,**F**). The figures on the left represent the relative abundance of genera and those on the right the relative abundance of species.

At 24 h, the identified phyla were Firmicutes (39%), Proteobacteria (29%) and Bacteroidota (20%); at the genus level the category “others” comprised 23% (referings to genera in non-significant percentages, but which added together gave this value), followed by the generas *Prevotella* (15%), *Streptococcus* (14%), and *Acinetobacter* (13%), then *Enterobacter* and unassigned genera (8%); followed by *Staphylococcus* (5%) (Figure 2A). At the species level *A. baumanni* (12%) was the predominant species identified, this corresponds to an intrahospital germ (Figure 2B). At the second point of time, 48 h after the first sampling, the phyla Proteobacteria, Firmicutes and Bacteroidota predominate with 34, 29 and 21% respectively. The most abundant genera were *Acinetobacter* with 19% *Prevotella* 14%, *Streptococcus* 13% and *Enterobacter* with 7% (Figure 2C). The most abundant species of the predominant phylum was *A. baumannii* (19%) (Figure 2D). At the third time point, at 72 h, the *Proteobacteria* phyla predominated with 41% followed by *Firmicutes* and *Bacteroridota* with similar percentages between 24 and 22%. At the genus level *Acinetobacter* reached occupies 18%, *Prevotella* 15%, *Enterobacter* 12% (Figure 2E) and *Streptococcus* 9%. *A. baumannii* continueds to be the predominant species, at 18% (Figure 2F).

**Figure 3:**
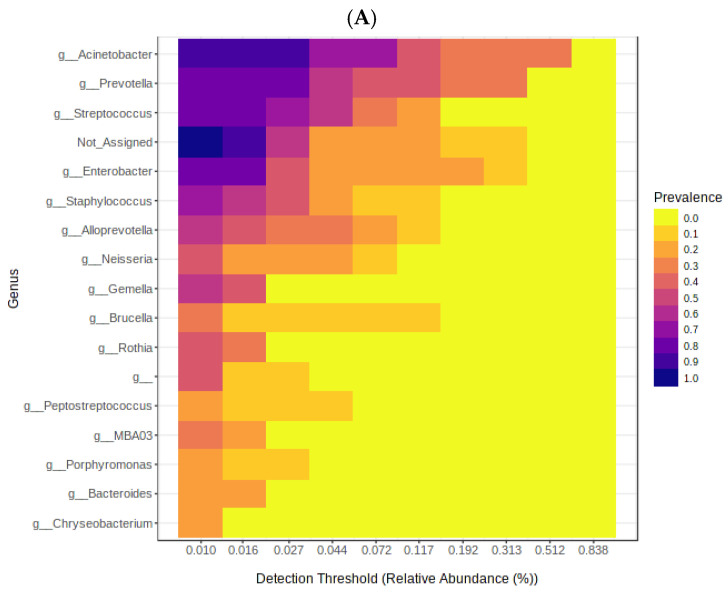

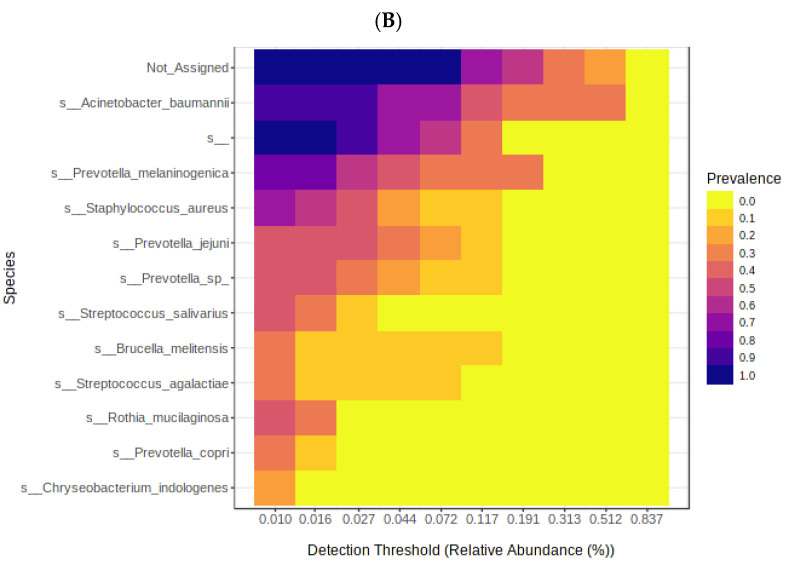
Heat map of the microbiome in surviving patients. (**A**) shows the most representative genus and (**B**) shows the most representative species.

The second classification was made by the condition at discharge from the unit. The first group, “surviving patients” showed Proteobacteria, Bacteroidota and Firmicutes as predominant with 47, 25 and 18% respectively. At the genus level, with 28% *Acinetobacter* was dominant followed by *Prevotella* 17% and *Enterobacter* 9%. In the last step, at the species level, 28% were *A. baumannii* and 27% were not assigned. According to the legend of the heat maps, a higher prevalence of the genera *Acinetobacter*, *Streptococcus*, *Prevotella* and *Enterobacter* (Figure 3A) and the species *A. Baumanni* and *P. melaninogenica* (Figure 3B) was found.

**Figure 4:**
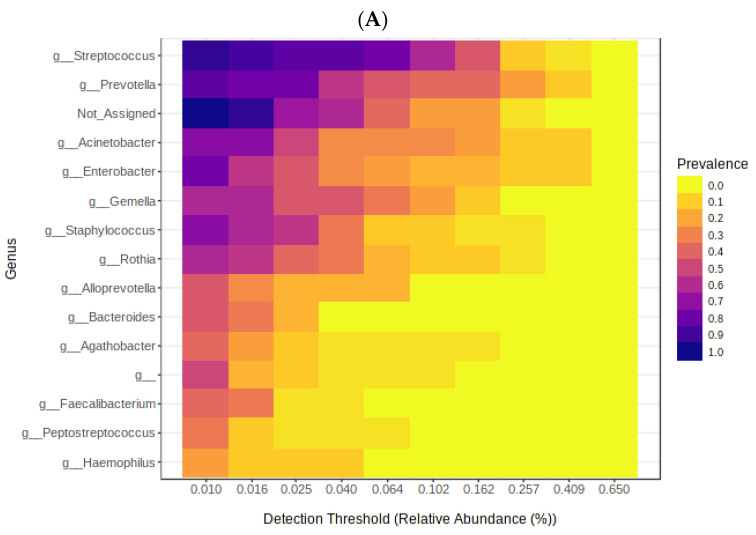

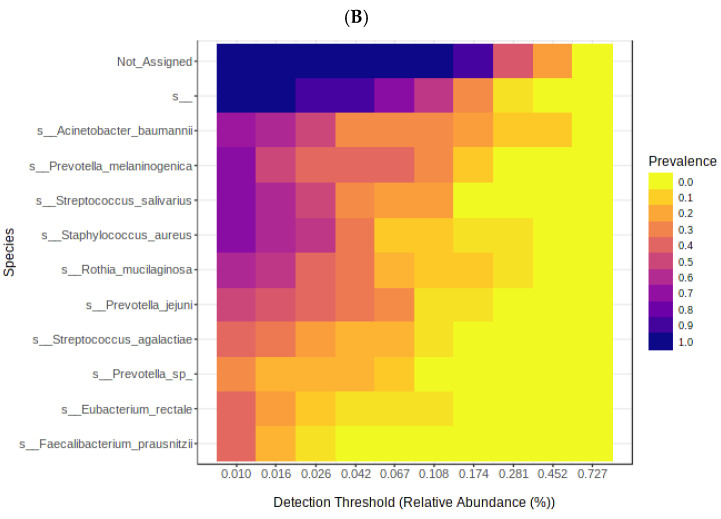
Heat map of the microbiome in deceased patients. (**A**) shows the most representative genus and (**B**) shows the most representative species.

In the second group “deceased patients”, the phyla that predominated were Firmicutes, Proteobacteria and Bacteroidota at 37, 28 and 19% respectively. At the genus level *Streptococcus* reached 15%, *Prevotella* 15%, *Acinetobacter* 11% and *Enterobacter* 9%. At the species level, *A. Baummanii* predominated with 11% and *P. melaninogenica* with 6%. The heat maps show a higher prevalence of the genera *Streptococcus*, *Prevotella* and *Enterobacter* (Figure 4A) while at the species level *S. salivarius*, *Staphylococcus aureus* and *P. melaninogenica* were predominant followed by *Acinetobacter baumannii* (Figure 4B).

### 3.2. Rarefaction Curves

**Figure 5:**
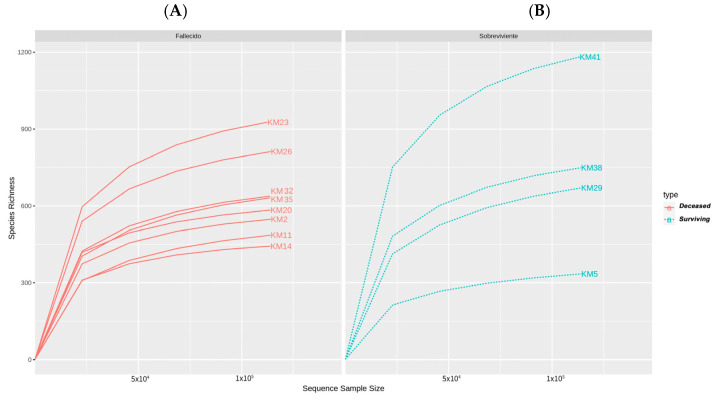
Rarefaction curve using the original data set. Groups: (**A**) Deceased (**B**) Surviving.

By this method, the relationship between the number of OTUs and the number of sequences is presented. According to these curves the deeper into the sequence or sample data you delve, the more species you will observe, until the point where you have observed all species in the sample. In this case, the number of reads and the depth of sequencing were sufficient to cover the microbial composition of the samples, as can be seen by the trend of the curves (Figure 5).

### 3.3. Alpha Diversity of Samples

**Figure 6:**
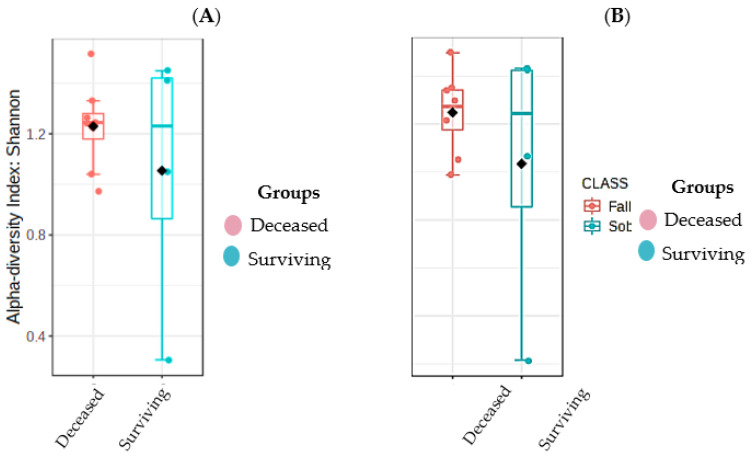
Diversity of the microbiota. (**A**) Alpha diversity according to the Shannon index of patient groups: survivors and deceased. (**B**) Alpha diversity according to the Simpson’s index of both groups.

Regarding the alpha diversity of the samples, the first index was the Shannon index which evaluates the diversity of the sample, higher values are more diverse. The surviving patients showed a Shannon index with a mean of 2.923219843, while the deceased patients showed a mean of 2.765490056. The sample with the highest diversity corresponded to sample KM26 (deceased patient), and the sample with the lowest diversity was KM5 corresponding to a surviving patient. The Kruskal Wallis method was used as a non-parametric method, since the data did not follow a normal distribution (Figure 6), and no significant difference was found between the surviving and deceased groups (*p* = 1).

Simpson’s index, referring to the richness of organisms, was also not significant between groups, with a mean of 0.745078386 in the group of survivors and 0.820381761 in the group of the deceased, with the highest value in the sample KM26 0.926605245 (deceased patient) and the lowest value KM5 0.301049914 corresponding to a surviving patient. According to the same statistical method, no significant difference was found between the two groups (*p* = 1).

### 3.4. Beta Diversity among Groups

**Figure 7:**
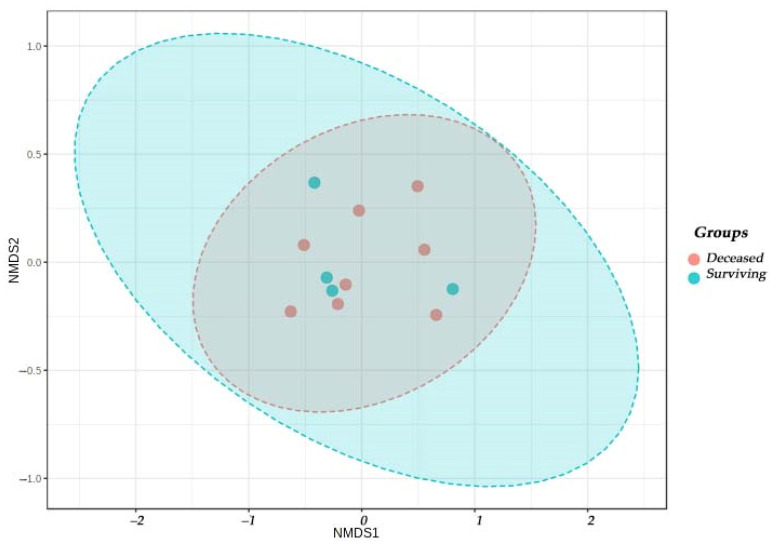
Beta diversity between in the groups of surviving and deceased patients.

The Beta diversity shows the variation between groups. This was performed according to the Bray Curtis similarity analysis, ANOSIM index. The R test statistic, (difference between mean ranks of groups and within groups, R-value −1 to 1) showed, no significant differences between the surviving and deceased groups, (*p* = 0.1387) (Figure 7).

## 4. Discussion

This prospective observational study described the composition of the microbial communities in a group of critical patients who were mechanically ventilated, for various reasons except for those with respiratory illnesses or with a history that could alter the microbiota. The aim was to find the relationship between the microbial profile and the severity of the disease and subsequent death. This study was based on the first studies of the microbiome in patients in intensive care units [23,24,25,26] and aimed to obtain a close approximation of the microbial composition in the environment, since there are no similar studies in critical patients at present. However, due to the context of COVID-19, this study had the limitation of not having a control group of healthy patients, so we instead focused on the differences between groups.

Christine M. Bassis in her study “Microbiota of the upper respiratory tract as the origin of the pulmonary and gastric microbiota in healthy individuals” identified Bacteroidotas, Firmicutes and Proteobacteria as the most common bacterial phyla [3], similar to Eduard Monsó who referred to these same phyla, but added that there was a low frequency of OTUs corresponding to potentially pathogenic microorganisms such as those of the genus *Haemophilus*. The identified edges coincided with those of our patients [27].

Analyses were carried out at three time cuts. At 24 h, we found the more abundant genera were *Prevotella*, *Streptococcus* and *Acinetobacter*. This composition varied at 72 h to *Acinetobacter*, *Prevotella*, *Streptococcus* and unassigned genera. A decrease in the genus *Prevotella* and an increase in ICU nosocomial germs such as *Acinetobacter* and *Enterobacter*, coincided with finding from the conventional cultures of the microbiological map of the unit (Figure 7). Similar results were found by Daphnée Lamarche in her study, indicating the loss of of commensal microorganisms during critical illness [28].

In another study by Min-gyung Baek, an analysis of two groups of patients on mechanical ventilation comprised of older adults from nursing homes with pneumonia compared with a group without pneumonia, they found that the average abundance of the genera *Corynebacterium*, *Staphylococcus* and *Pseudomonas* was higher in the pneumonia group than in the non-pneumonia group while the relative abundances of *Streptococcus* and *Prevotella* were higher in the non-pneumonia group. It should be noted that these two genera ranked second and third in abundance in our study. The exclusion criteria included non-pulmonary respiratory failure. On the other hand, in healthy individuals, Christine M. Bassis distinguished the following genera in greater abundance: *Prevotella*, *Veillonella* and *Streptococcus*. Likewise, in another study carried out by Daphnée Lamarche, *Veillonella*, *Prevotella* and *Neisseria* denominated in healthy patients in the lower respiratory tract, varying the relative abundance in critically ill patients of *Enterococcus*, *Pseudomonas* and *Staphylococcus*, germs that are related to nosocomial infections [28].

In relation to other pathologies, the lung microbiota has been extensively studied in chronic respiratory diseases such as COPD and, cystic fibrosis and also in the role it could play in acute pathologies such as pneumonia or ventilator-associated pneumonia. Alison J. Dicker 2020, in a study on COPD, identified Proteobacteria and Firmicutes as the predominant phyla, with fewer OTUs identified for *Bacteroidetes*, *Actinobacteria* and *Fusobacteria*. At the genus level, the most abundant genera were *Haemophilus*, *Streptococcus*, *Neisseria*, *Veillonella* and *Prevotella*. *Proteobacteria dominance* was associated with higher mortality compared to *Firmicutes*-dominated or balanced microbiome profiles [29]. On the other hand, in a study by Robert P. Dickson on patients with Acute Respiratory Distress Syndrome (ARDS), the lung microbiota was characterized by enrichment of the Proteobacteria phylum and this was significantly associated with elevated alveolar concentrations of tumor necrosis factor alpha (TNF-α), a key mediator of lung inflammation in ARDS that is independently predictive of mortality. A decrease in TNF-α was associated with the enrichment of *Bacteroidetes*, the most abundant edge in the lung of the healthy subject; In a similar case, in a study by Alison J. Dicker 2021, in a group of patients with bronchiectasis (both stable and with exacerbations), a microbiome dominated by *Proteobacteria* and *Firmicutes* was found [30].

Taking the results together, a difference can be seen in the abundance of *Bacteroidota*, with a percentage of 25% in survivors and 19% in the group of patients who died. Similarly, over time, *Proteobacteria* increased from 29 to 34% at 48 h, becoming the majority with 41% at 72 h. Within this phylum are the highly negative nosocomial bacteria that we found in our study, such as *A. baumannii* complex/haemolyticus, *K. pneumoniae*, and *Enterobacter cloacae*, and the increase in this phylum could be due to colonization and/or infection in these patients.

A slight difference was observed in favor of the surviving patients when evaluating the diversity within the sample compared to the group of deceased patients (however, it was not significant). A similar result was found in the study by Johnstone et al. [28] but here the decrease was significant in the respiratory microbial diversity in patients who died versus those who survived their stay in the hospital. In addition, several studies have established that the microbial composition tends to be dominated by only a few taxa [31,32].

The results of diverse abundance among the samples showed that the occurrence of microorganisms is specific to each patient. It is also important to note that, at the genus level, a notable percentage was not assigned, probably because Shotgun metagenomics was not used and regions of the 16S rRNA gene were sequenced, which allowed for greater specificity at the phylum level.

The loss of diversity makes sense since this decrease in diversity was observed only with parameters such as the Shannon and Simpson diversity index. These results suggest the likely use of α-diversity metrics as an index of disease severity and could, along with other clinical indicators, improve the classification of patients based on their survival prognosis [33].

Samples were taken at a relatively early time (at 48 h), so the results were not associated with antibiotic administration, however, in other studies, no correlation between antibiotic administration and microbial diversity has been observed, indicating that much depends on the time of sampling [28,34,35].

Studies such as that of Brendan et al. took samples at different time points, showing that the patients had a lower initial diversity at both sites and that this diversity decreased even more with time on the ventilator [22]. Other authors such as Zakharkina et al. concluded that mechanical ventilation, but not antibiotic administration, was associated with changes in the respiratory microbiome. The dysbiosis of microbial communities in the respiratory tract was more profound in patients who developed ventilator-associated pneumonia [35]. A similar study to ours by Huebinger et al. took samples from a small group of patients in a surgical ICU, so the need for ventilation in this cohort was mainly attributed to traumatic injury. It is reasonable to assume that the common microbiome identified here may be similar to that of a healthy individual, and they were unable to determine the effects of antibiotics on the lung microbiome profile [36].

Our results allow us to expand the knowledge of the lung microbiome of a critically ill patient in order to find biomarkers for the diagnosis and prognosis of respiratory diseases. On the other hand, if a microbial profile of a critical vs. healthy patients is established, it would be much easier to modify the microbiota than to modify the genetic predisposition of a patient and in this way obtain a more effective response to the treatment.

## 5. Conclusions

The present study showed that with data obtained by 16S rRNA gene sequencing and the identification of a microbial profile that was much more accurate than that offered by conventional cultures we may obtain valuable information for patient prognosis. However, performing such analysis with more representative samples is of vital importance to comfirming these differences and to make meaningful comparisons.

The genus *Prevotella* had less abundance in deceased patients than in survivors. The Shannon index was inversely correlated with the APACHE II and the days of stay in the ICU were significantly related to mortality.

It was not possible to identify a characteristic profile in this group of patients as a predictor of mortality; however, there is a decrease in commensal bacteria in the deceased patients and a progressive increase in in-hospital germs. The abundance of pathogens within 24 h of intubation was striking. Studies with larger numbers of patients are needed to define significant associations.

Despite this, a difference in the microbial composition was noted, with different phyla predominating, associated with mortality was observed between groups. The role that characterization of the microbial profile could play will be important in further research on factors associated with mortality.

On the other hand, our results contribute to the increase in evidence to determine that the airway microbiome plays an important role in lung disease and its response to treatment; however, the lack of studies will make it difficult to fully understand that eubiosis and dysbiosis really exist in the lung microbiome.

## Figures and Tables

**Table 1:** Demographics characteristics of the mechanically ventilated critically ill patients.

Patient	Sex	Age	Reason for Admission			Comorbidities	Days of Mechanical Ventilation	Days of Stay in UCIE	Condition at the Termination of the VM	APACHE II Score	SOFA Score	21 Day Survival	28 Day Survival
			Medica	Surgical	Traumatic								
A	F	67	Acute pancreatitis	-	-	^2^ HBP, ^3^ T2DM	10	10	D	24	11	No	No
B	F	49	-	-	Closed abdominal polytraumatized	None	17	20	S	15	4	Yes	Yes
D	F	75	-	Mesenteric thrombosis	-	None	24	24	D	23	6	Yes	No
E	M	67	-	Mesenteric thrombosis	-	HBP, Hepatic cirrhosis	16	16	D	23	7	No	No
G	M	51	-	-	Polytraumatize, severe ^1^ TBI	HBP	11	11	D	20	9	No	No
H	F	72	-	Gastric perforation	-	HBP, ^4^ CKD-HD, ^5^ Afib with RVR, Barrett’s esophagus	7	7	D	24	10	No	No
I	M	81	-	-	Severe TBI, subdural hematoma	T2DM	23	23	D	22	11	Yes	No
J	M	65	-	-	Severe TBI	HBP	6	7	S	15	8	Yes	Yes
K	F	77	-	Sigmoid volvulus, intestinal obstruction for sigmoidectomy	-	None	11	11	D	17	6	No	No
L	F	77	-	Subarachnoid hemorrhage	-	Hepatic cirrhosis	11	11	D	28	10	No	No
M	M	60	-	-	Subdural hematoma, parietooccipital lamellar	HBP, CKD-HD	23	25	S	18	11	Yes	Yes
N	M	49	-	Hemorrhagic stroke, aneurysm clipping	-	None	6	9	S	17	5	Yes	Yes

Note: F: female, M: male, D: desease, S: Survivor. ^1^ traumatic brain injury ^2^ high blood pressure ^3^ type-2 diabetes mellitus ^4^ chronic kidney disease on hemodialysis, ^5^ atrial fibrillation with rapid ventricular response.

## Data Availability

Data are contained within the article.
